# Whole-Genome Sequences of SARS-CoV-2 Isolates from the Dominican Republic

**DOI:** 10.1128/MRA.00952-21

**Published:** 2021-11-24

**Authors:** R. Paulino-Ramirez, E. Riego, A. Vallejo-Degaudenzi, V. V. Calderon, L. Tapia, Patricia León, Danilo Licastro, Simeone Dal Monego, Sreejith Rajasekharan, Emanuele Orsini, Alessandro Marcello

**Affiliations:** a Instituto de Medicina Tropical & Salud Global, Universidad Iberoamericana, Santo Domingo, Dominican Republic; b Molecular Biology Department, Referencia Clinical Laboratory, Santo Domingo, Dominican Republic; c AREA Science Park, Trieste, Italy; d Laboratory of Molecular Virology, International Centre for Genetic Engineering and Biotechnology, Trieste, Italy; DOE Joint Genome Institute

## Abstract

Here, we report the genome sequences of five severe acute respiratory syndrome coronavirus 2 (SARS-CoV-2) strains that were obtained from symptomatic individuals with travel histories during community surveillance in the Dominican Republic in 2020. These sequences provide a starting point for further genomic studies of gene flow and molecular diversity in the Caribbean nation. Phylogenetic analysis suggests that all genomes correspond to the B.1 variant.

## ANNOUNCEMENT

Coronaviruses have emerged in the past century, causing epidemics and pandemics of zoonotic strains (1). Coronavirus disease 2019 (COVID-19), the disease caused by severe acute respiratory syndrome coronavirus 2 (SARS-CoV-2), a member of the family *Coronaviridae*, genus *Betacoronavirus*, has caused a global pandemic with unprecedented impact on humans and has highlighted the weaknesses of the response systems for health emergencies in developing countries (2).

**FIG 1:**
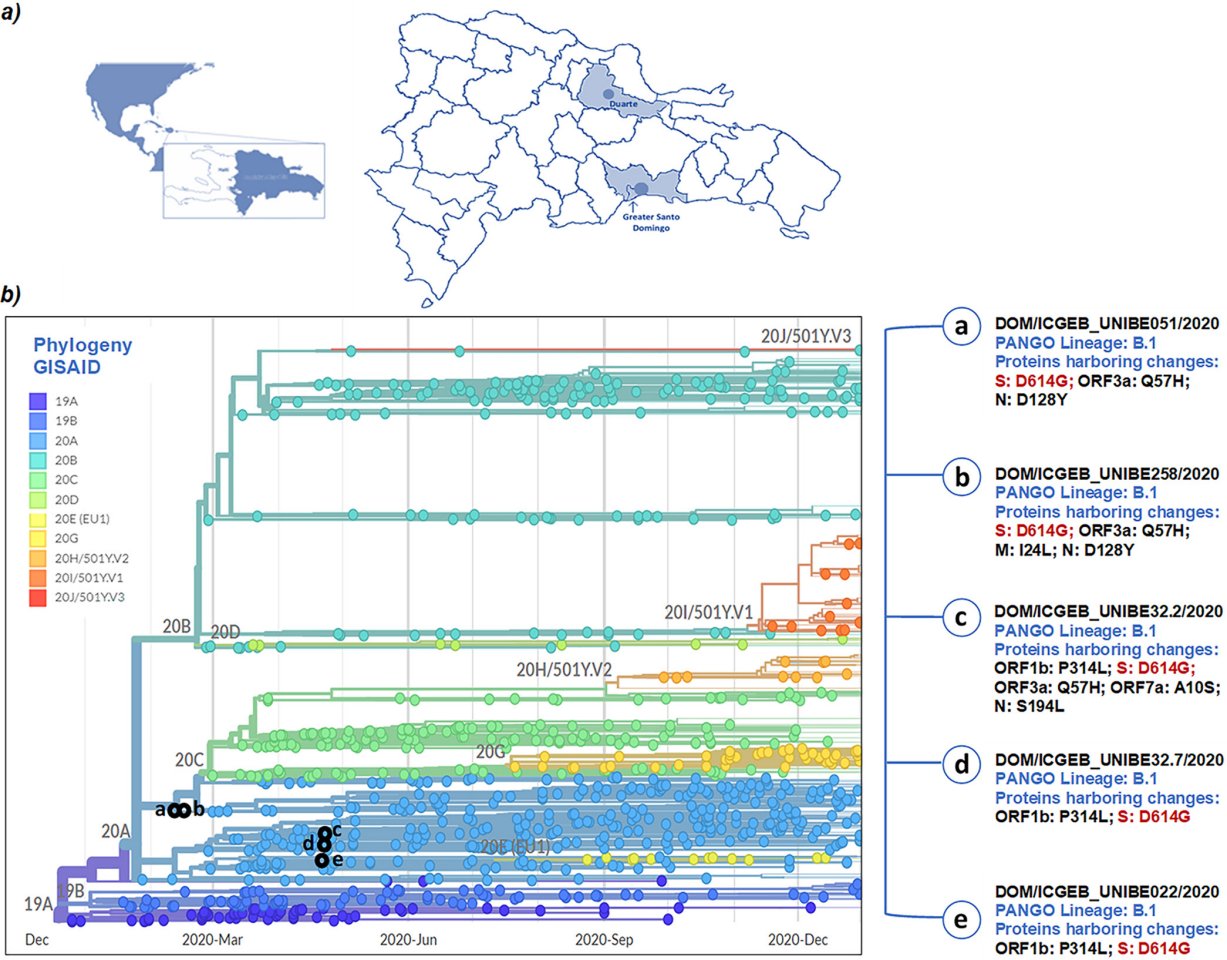
(A) Map showing the island of La Hispaniola in the Caribbean basin (right), the Dominican Republic (dark blue), and localities (dark blue circles) where samples were collected in provinces in blue, i.e., Duarte (located in the north-central plateau) and Greater Santo Domingo (on the southern coast), which includes the capital city (Distrito Nacional) (arrow) and the nearby municipalities. Maps were generated under the ArcGIS software v10.8.1 license. (B) Phylogenetic analysis of SARS-CoV-2 including five genome sequences collected in the Dominican Republic. Available genomes were retrieved from GISAID (https://www.gisaid.org) on 1 September 2021 but were limited to December 2020 to better allocate the early sequences detected. Colors depict clades based on mutation marks using the GISAID standardized nomenclature. All analyzed sequences were classified as B or B.1 (PANGO Lineage), harboring S protein changes in the 614 codon (D614G) (in red); unique mutations in S protein are highlighted in blue.

After the first case was identified in Wuhan, China, in late December 2020, the virus was first reported in La Hispaniola on 28 February 2020, with an expanding wave of transmission all over the Dominican Republic (3–5). The first cases in the country were detected in Greater Santo Domingo (which includes the capital city and the neighboring province) and San Francisco de Macoris, a north-central town in the Duarte Province (Fig. 1A) (6–8).

A total of five samples were collected from symptomatic individuals with travel histories during community surveillance in February to May 2020. Cases were associated with a history of travel to Italy and were geographically from a north-central community (in the Duarte Province) and Greater Santo Domingo (Fig. 1A), representing the first confirmed SARS-CoV-2 cases in the country. Samples were collected using nasopharyngeal swabs. RNA extraction was performed using the MagMax viral/pathogen nucleic acid isolation kit in the KingFisher Flex automated extraction system (Thermo Fisher Scientific) following the manufacturer’s protocols. For each sample, 100 ng of total RNA was processed using the Zymo-Seq RiboFree ribosomal depletion library preparation kit (Zymo Research) (9). A Qubit 2.0 fluorometer (Thermo Fisher Scientific, MA, USA) and Agilent 2100 Bioanalyzer (Agilent Technologies, CA, USA) were used to assess RNA quantity and quality. Total RNA was processed using library construction based on the Swift Amplicon SARS-CoV-2 research panel (Swift Biosciences, USA), which provides optimal coverage and data quality. High-throughput sequencing was conducted using an Illumina MiSeq sequencer following the standard procedure. The raw sequence data were quality controlled using FastQC v0.11.9 37 (https://www.bioinformatics.babraham.ac.uk/projects/fastqc). Genome assembly was conducted using dedicated Swift guidelines (10); the genome size, coverage depth, and overall GC content of each genome are indicated in Table 1 (11). All tools were run with default parameters unless otherwise specified.

**TABLE 1:** Bioinformatic details of analyzed sequences

Parameter	Finding for strain:				
	ICGEB_UNIBE32.7	ICGEB_UNIBE051	ICGEB_UNIBE258	ICGEB_UNIBE022	ICGEB_UNIBE32.2
SRA accession no.	SRX12761935	SRX9816443	SRX9816442	SRX9816441	SRX9816440
GISAID clade	O	GH	GH	G	GH
No. of raw reads	929,390	893,092	76,794	1,203,034	1,070,364
Genome size (bp)	29,585	29,901	29,580	29,902	29,748
SARS-CoV-2 coverage depth (×)	2,761	5,000	83	5,447	567
GC content (%)	48.7	44.8	45.6	43.5	46.2

Phylogenetic tree analysis was conducted using the Nextstrain bioinformatics platform (http://nextstrain.org/ncov) with the maximum likelihood option and the JTT matrix (12–15). The complete genome sequences were analyzed in the context of the Nextregions/North American data set, which is available at the GISAID site (updated to 1 September 2021). A Dominican Republic-focused country-level subsampling strategy was performed, using the reference strain hCoV-19/Wuhan/WH01/2019 (GISAID accession number EPI_ISL_402125) as the original root. Figure 1B shows the genetic relationship between Dominican samples and other strains in the GISAID database. This study represents the starting point to further explore the genomic diversity of SARS-CoV-2 in the Dominican Republic, since its introduction, human mobilization across the terrestrial border with Haiti, and the tourism industry.

The institutional review board at Universidad Iberoamericana (UNIBE) (CEI-2020-16) and the National Bioethical Committee (020-2021) approved this study.

### Data availability.

Sequences were deposited in the NCBI database (BioSample accession numbers SAMN17274443, SAMN17274444, SAMN17274445, SAMN17274446, and SAMN22555599). The raw reads were deposited in the NCBI Sequence Read Archive (SRA) database (SRA accession numbers SRX12761935, SRX9816443, SRX9816442, SRX9816441, and SRX9816440) under BioProject number PRJNA691021. Genome sequences were deposited in the NCBI GenBank database (GenBank accession number OK523387, OK523388, OK523389, OK523390, and OK542388) and in the GISAID database (GISAID accession numbers EPI_ISL_523811, EPI_ISL_523812, EPI_ISL_525467, EPI_ISL_525468, and EPI_ISL_525469).
